# Role of Epithelium-Derived Cytokines in Atopic Dermatitis and Psoriasis: Evidence and Therapeutic Perspectives

**DOI:** 10.3390/biom11121843

**Published:** 2021-12-07

**Authors:** Francesco Borgia, Paolo Custurone, Lucia Peterle, Giovanni Pioggia, Sebastiano Gangemi

**Affiliations:** 1 Department of Clinical and Experimental Medicine, Dermatology, University of Messina, Via Consolare Valeria—Gazzi, 98125 Messina, Italy; paolo.custurone@gmail.com (P.C.); lucia.peterle@gmail.com (L.P.); 2 Messina Unit, Institute for Biolomedical Research and Innovation, National Research Council of Italy (IRIB-CNR), Via Vincenzo Leanza, Mortelle, 98164 Messina, Italy; giovanni.pioggia@irib.cnr.it; 3 Department of Clinical and Experimental Medicine, School and Operative Unit of Allergy and Clinical Immunology, University of Messina, Via Consolare Valeria—Gazzi, 98125 Messina, Italy; sebastiano.gangemi@unime.it

**Keywords:** psoriasis, atopic dermatitis, TSLP, interleukin 25, interleukin 33, epithelium derived cytokine, skin, inflammation, oxidative stress, biological therapy

## Abstract

Atopic dermatitis and psoriasis are two of the most common chronic skin conditions. Current target therapies represent viable and safe solutions for the most severe cases of these two dermatoses but, presently, several limitations exist in terms of efficacy and side effects. A new class of products, epithelium-derived cytokines (TSLP, IL-25, IL-33), show an increasing potential for use in target therapy for these patients, and demonstrate a direct link between a generalized inflammatory and oxidative stress status and the human skin. A review was conducted to better understand their role in the aforementioned conditions. Of these three molecules, TSLP led has been most often considered in studies regarding target therapies, and most of the results in the literature are related to this cytokine. These three cytokines share common stimuli and are linked to each other in both acute and chronic phases of these diseases, and have been challenged as target therapies or biomarkers of disease activity. The results lead to the conclusion that epithelium-derived cytokines could represent a therapeutic opportunity for these patients, especially in itch control. Furthermore, they might work better when paired together with currently available therapies or in combination with in-development treatments. Further studies are needed in order to verify the efficacy and safety of the biologic treatments currently under development.

## 1. Introduction

Currently, inflammatory dermatoses pose a serious challenge for dermatologists worldwide. Two of these, atopic dermatitis and psoriasis represent a daily reminder for the patients and practitioners of the current limitations of treatments and understanding of their pathophysiology. Psoriasis (Pso), characterized by erythematous, scaly patches on the skin and affecting other body districts such as bones and accessory skin structures, presents comorbidities with seemingly unrelated non-skin conditions such as a metabolic syndrome, heart disease and psychiatric disorders [1,2]. Lately, Pso has also been described as an inflammatory disorder in which oxidative stress, advanced glycosylation end-products (AGEs) and advanced oxidation protein products (AOPP) play a major role in cytokine secretion [3]. On the other hand, atopic dermatitis (AD) consists of erythematous patches scattered over specific areas of the body which define its subtype and a constant, intensity-variable pruritus, which is enough to lower the overall quality of life of the patient [4,5]. Lately, this disease has also been pointed out as heavily oxidative stress-related, since production, dismission and dosage of AGEs and AOPPs present increasingly interesting results [6]. Both these diseases share a complex interlinking of external and internal factors in the pathogenetic processes, in a strongly represented inflammatory aspect in the skin samples collected so far, which leads to their manifestation, and both share a leading role played by cytokines [7,8]. Three cytokines have been researched in recent years regarding possible physiopathology links and therapeutic strategies, namely, Interleukin 25 (IL-25), interleukin 33 (IL-33) and Thymic Stromal Lymphopoietin (TSLP). IL-25 belongs to the IL-17 family and is known as IL-17E. This cytokine has proven to play a pivotal role in many inflammatory diseases such as spondylarthritis, inflammatory bowel disease and cancer but has also been found in Th2-oriented responses, particularly in asthma [9]. Through the production of this cytokine, responsive cells such as Th2-oriented immune system cells, natural killer lymphocytes (NKT) and inherent lymphoid type 2 cells (ILC2) produce allergy related interleukins thus perpetrating the inflammatory processes [9]. The IL-33 cells, affecting Th2 cells and NKTs, acts also as a chemokine and activates other immune cells such as neutrophils and macrophages, thus suggesting a more pleiotropic role than IL-25 [10]. TSLP is a cytokine produced by stromal and endothelial cells after cellular damage and allows the maturation of dendritic cells and release of chemokines by monocytes [11], so far found to be involved in the pathogenesis of several Th2-oriented diseases, in particular asthma. Overall, these three cytokines are collectively known as epithelium-derived cytokines, acting as alarmins after a proper, external stimulus acts upon the skin and mucosae, triggering a Th2 response from the body. The studies listed in this literary review postulate that these cytokines might be involved in other conditions, where the response is chiefly represented by Th1/Th17 cells, such as in psoriasis. The articles discussed in this research suggest a possible role of monoclonal antibodies as future treatments for these diseases, possibly covering any undergoing mechanisms that are not already targeted by available biologic medications. Given these factors, the aims of this review are as follows: (1) Evaluate current knowledge about epithelium-derived cytokines in the pathogenesis of two chronic, inflammatory skin conditions such as Pso and AD; (2) Suggest possible links to Th1, Th2 and oxidative stress responses in the most severe cases of Pso and AD; (3) Correlate levels of these cytokines to severity, phenotype and treatment response in Pso and AD; (4) Suggest possible therapeutic options in light of newfound discoveries. Here, we provide numerous articles found through this research. For commodity reasons, they have been subdivided based on the interleukin considered (IL-25, IL-33, TSLP) and by pathology (Pso, AD). For each cytokine, three tables have been submitted at the end of this chapter—Table 1 for IL-25 and skin, Table 2 for IL-33 and skin, Table 3 for TSLP and skin.

## 2. Psoriasis: Role of Epithelium Derived Cytokines in Skin Damage

### 2.1. IL-25 and Psoriasis

Xu et al. found that IL-25 levels are increased in the psoriatic skin of mice specimens. In this model, knockout mice for the gene that encodes for IL-25 showed lower amounts of acanthosis, dermal thickness and immune cell infiltration, while injection of IL-25 alone led to the appearance of psoriatic lesions [12]. Senra et al. found that IL-25 leads to an accumulation of neutrophils led by macrophages in the site of injection of mice specimens. The psoriasis-like lesions disappeared after five days from the last injection, suggesting their acute role in the development of skin inflammation. The blockade of IL-25 through specific antibodies led to the result of turning off inflammation as well [13]. Borowczyk et al. found that IL-22, a cytokine mainly involved in epithelial and stromal cells, can upregulate the production of IL-25, which, as an autocrine effect, leads to proliferation and spikes in metabolic activity in vitro [14]. Senra et al. found that, in Pso lesions, levels of IL-25+ cells were higher than those found in control specimen, in particular in the lowermost levels (basal and supra-basal) of the epidermis and activating M2 macrophages to recruit neutrophils where the abscesses of Munro should develop (lowermost strata of epidermis) [15].

### 2.2. IL-33 and Psoriasis

Chen et al. found that patients with moderate–severe psoriasis have both sera- and intra-epidermal levels of Il-33 higher than healthy controls and, in the same study, an Imiquimod induced Pso model in experimental mice was ameliorated by the injection of IL-33, suggesting the possible anti-inflammatory role of this cytokine [21]. Borsky et al. investigated the levels of various alarmins among which IL-33 in the serum of psoriatic patients and they found that its levels were significantly higher than in the controls but did not corelate with the severity of the disease [20]. Meephansan et al. proposed two branches of treatment in Pso patients, treated with methotrexate (MTX) or treated with narrow-band ultraviolet rays B (NB-UVB). In the group treated with MTX, levels of IL-33 were lower than before treatment; in contrast, after UVB therapy, levels increased. This led to contrasting results, since the shutting down of IL-33 by MTX led to an amelioration of the skin patches, but UVB therapy alone might produce a direct increase of this particular cytokine as a countereffect, without involving it in the pathogenetic process. The study did not suggest any other interpretations [19]. Raimondo et al. demonstrated that IL-33 from psoriatic plaques induces the release of a wide range of osteoclastogenic factors from the skin, such as RANKL, that promote monocyte differentiation in osteoclasts and other osteoclastogenic mediators that could act in a RANKL independent pathway. These results suggest a link between psoriatic cutaneous inflammation and the pathogenesis of psoriatic arthritis (PsA), thus explaining why in most cases Pso anticipates several decays in the onset of PsA [18].

### 2.3. TSLP and Psoriasis

Gago-Lopez et al. found that TSLP, in psoriasis, plays a wide variety of roles; TSLP enhances cell proliferation (via STAT5 pathway activation) and angiogenesis (via vascular endothelial growth factor production). In a murine model, antibodies directed against TSLP led to a positive response in terms of epidermal thickening and vascularization [38]. Tashiro et al., using cultures of HaCaT cells (immortalized keratinocytes), found that TSLP, in hypoxic conditions, presents reduced levels via the inactivation of its production stimulated by TNF-alpha, via HIF2 and HRE mechanisms (two products involved in the skin response to hypoxemia). It is the authors’ suggestion that targeting these two molecules could lead to lower levels of TSLP and the resolution of lesions both in Pso and AD [37]. Schaper et al. correlated the levels of TSLP from human skin samples derived from healthy controls, Pso patients and AD patients, noticing that both Pso and AD patients, but mostly Pso patients, presented higher levels of TSLP, especially the inflammation-related isoform rather than the basal one, via histamine release by hyper-active Th2 cells [34]. Segawa et al. developed a model using HaCaT cells again, demonstrating that EGFR is transactivated via TSLP by TNF-alpha; it is the author’s suggestion that, since anti-TNF drugs may produce many side effects (such as infections), targeting TSLP instead of TNF-alpha could provide the same results with greater benefits [35]. Chen et al., in a 2021 study, matched healthy controls to AD and Pso patients, showing that it is possible to differentiate two separate groups of Pso patients; the most interesting is the “early onset” group, with high levels of Th2 cells and a high TSLP response. The levels of TSLP were higher than those present in AD, suggesting that different subtypes of Pso could benefit from different types of treatment [39]. Desmet et al. suggested a novel approach in their study, in which they tried to use RNA interfering molecules, applied topically, to silence the production of several mRNAs among which the mRNA affecting TSLP release. As a result, levels of TSLP dropped, along with the other products, suggesting a possible future treatment for Pso in a 3D model skin [36].

## 3. Atopic Dermatitis: Epithelium Derived Cytokines and Their Role in Damage Propagation

### 3.1. IL-25 and Atopic Dermatitis

De Vuyst et al. demonstrated that after incubation with a molecule that disrupts the membrane of the cells, after allowing the cells to recover from damage, there were no significant histologic changes, whilst if incubated with IL-25 while damaged, there were spongiosis and hyper-granulosis, two typical features of AD [17]. Yi et al. found that Intelectin, a lectin linked to the innate immune response, presents higher levels in skin samples of calcipotriol-induced AD. Along these findings, levels of IL-33 and TSLP, but not IL-25, were upregulated, thereby suggesting a possible role of the innate immune system in the pathogenesis of AD lesions [16].

### 3.2. IL-33 and AD

Pietka et al., to investigate the role of IL-33, which is a well-known factor involved in the pathogenesis of AD, proposed a calcipotriol-induced AD model in mice skin. The work group noticed that both wild type and knockout mice for IL-33 and its receptor developed AD lesions after treatment with calcipotriol, with no difference either macroscopically or microscopically. In addition, they did not find significant differences in the expression il Th2 cytokines between the two groups [33]. Salamon et al. demonstrated that IL-33 induces a production of IL-2, a mast cells (MC) derived interleukin involved in the suppression of chronic dermatitis, from isolated murine bone marrow derived-mast cells (BMMCs). In addition to this, they found that the mitogen-activated protein kinases (MAPKs) JNK and p 38, but not ERK, are crucial for IL-33 induced MC production of IL-2. Ultimately, they demonstrated that IL-2 released by IL-33 stimulation leads to an expansion of regulatory T cells (Tregs), which is fundamental in limiting the inflammatory response in AD [22]. The role of IL-33, however, does not appear univocal. In a murine model of AD developed by Sawada et al. indomethacin-induced lesions produced an increase of both levels of IL-33 and TSLP, but only in the case of mice with deficient levels of the TSLP receptor was there a less severe level of inflammation when challenged with this irritant. These findings suggest the potentially more prominent role of TSLP as opposed to IL-33 in the pathogenesis of this disease [28]. Dai et al. incubated both monolayers of cell cultures and human skin equivalents (HSE) in a medium rich in *Dermatophagoides pteronissynus* allergens, aiming to find a possible external stimulus for the endogenous production of IL-33. The authors found that these allergens, through the activation of metalloproteinases (MMP) and the epithelial growth factor receptor (EGFR), lead to an overexpression of this cytokine, leading to a Th2 response capable of inducing, in vivo, AD manifestations via the decreased production of filaggrin [32]. Ryu et al. developed a model of AD in normal human keratinocytes (NHEK) and HSE cultures by exposing the cells to higher concentrations of IL-33. They found that these cells showed a significantly lower concentration of the protein claudin-1, which are fundamental in tight-junctions and prevent water loss through the epidermal barrier [27]. Nygaard et al. investigated the role of IL-33 in the pathogenesis of AD in a 3D culture of keratinocytes. They demonstrated that after the incubation of this culture with IL-33, a downregulation of the expression of several genes from members of the epidermal differentiation complex occurs, including filaggrin. This result suggests a fundamental role of IL-33 in the atopic dermatitis pathophysiology, related to the negative regulation of structural proteins, stratum corneum formation and epidermal growth [24]. Chen et al. provided a phase 2 study of a single intravenous injection of etokimab, a humanized monoclonal antibody against IL-33 in adults with moderate to severe AD. In addition, they obtained skin samples after placebo and etokimab administration. The results suggest a good tolerability of etokimab, and a good profile of efficacy measured by the Eczema Area and Severity Index (EASI) score. In addition, they demonstrated a significant reduction of IFN-gamma serum levels, a significant reduction in peripheral eosinophil absolute counts 29 days after administration and a direct and IL-8 mediated reduction of neutrophils skin infiltration in samples of skin exposed to house dust mites [29]. Nakamura et al. evaluated IL-33 expression in the stratum corneum of the trunk skin by immunostaining adult patients with AD to correlate the skin levels of IL-33 with the severity of AD. They found that IL-33 expression was elevated in AD lesions and was significantly associated with the scores of lichenification and itching, suggesting that IL-33 plays an important role in the development of chronic lesions in AD [30]. Seo et al. studied the role of transient receptor potential vanilloid 3 (TRPV3), a member of the thermosensitive TRP channels in the development of AD. Primally, they found that TRPV3 levels, measured by Western blotting analysis in human keratinocytes cultures, derived from patients with AD and from an in vivo mice model of AD, are significatively higher and more activated than normal skin keratinocytes or keratinocytes derived from patients with other skin diseases. They demonstrated that the activation of TRPV3 can induce the production of IL-33, which plays an important role in the pathogenesis of the pruritus in AD, suggesting the use of the TRPV3 channel as a possible target therapy [31]. Jang et al. studied the role of the house dust mite (HDM) in the development of AD in in vitro and in vivo models of AD, and in samples derived from affected patients, demonstrating that HDM induces the release of IL-25 and IL-33 in epidermal keratinocytes. Using Toll-like receptor 1 (TLR1) and Toll-like receptor 6 (TLR6) knock-out mice, they demonstrated the role of these members of pattern recognition receptors (PRRs) in the release of IL-25 and 33 and in the activation of the Th2 response [23]. The role of IL-33 was studied by Tang et al. in a murine model of AD-like dermatitis, caused by the knock down of the molecule SHARPIN, a cell product that has scarcely been studied, which leads to a Th2 response and could constitute a new field of research for biological drugs. Indeed, IL-33 was found to be one of the main cytokines involved in the pathogenesis of this condition [25]. Peng et al. proposed a cure for AD using antibodies against the soluble form of IL-33, showing promising results in terms of lichenification, redness and scaling of the skin of the mice used in this model, with similar results when compared to topical tacrolimus administration [26].

### 3.3. TSLP and AD

Noh et al. studied the role of ZAG in AD, an adipokine involved in the mobilization of lipids, and its correlation with TSLP levels. They found lower levels of ZAG in the plasma, T-cells and in the skin of AD patients. Interestingly, the authors found that ZAG regulates TSLP secretion based on its efficacy in maintaining skin homeostasis and the barrier function [57]. Uysal et al. compared 60 AD-affected children and 31 healthy controls, evaluating the levels of TSLP in their sera. Higher levels of TSLP in the blood correlates to the severity of manifestations and it is the authors’ conclusion that it could be used, along with other markers such as periostin 1 and thymus and activation-regulated chemokine (TARC) as biomarkers in AD [44]. Barr et al. proposed a murine model of AD, induced by dinitrofluorobenzene, to evaluate the efficacy of a therapy based on PZ-235, a molecule developed to act as antagonist for the surface membrane receptor Protease activated receptor 2 (PAR2), with a reduced thickening of the induced lesions. In fact, one of the main effects of PAR2 is the upregulation of cytokine production belonging to the Th2 profile, in which TSLP represents one of the main elements [52]. Bogaczewicz et al. sought to better define the relation between inflammatory mediums in case of AD, demonstrating that levels of Th2 cytokines such as IL-5, TARC and TSLP are affected by each other. Their main finding, however, is represented by the fact that UV-A therapy did not result in a lowering of TSLP serum levels, suggesting that AD might represent a basal inflammatory status [40]. Lee et al. defined the relationship between TSLP and antimicrobial peptides (AMP) TSLP acts as a down regulator of AMPs via the JAK/STAT3 pathway, leading to a dramatically reduced barrier function and predisposition to developing microbial infections [41]. Gu et al., similarly to Lee, posited that, perhaps just indirectly, skin flora might represent a trigger for the development of AD since the presence of lipopolysaccharide (LPS) is a sufficient stimulus, and that bland topical treatments have the ability of downregulating the expression of TSLP [58]. Kitajima et al. proposed a mouse model to define a possible future cure for AD, suggesting that the blockade of the receptor of TSLP (TSLPR) in CD4+ lymphocytes interrupts the chronic phase of inflammation (but not the acute phase), which was proven using knock-out mice for this surface receptor [59]. Wallmeyer studied the effects of TSLP on a mice knock out model for the gene-encoding filaggrin, one of the main factors involved in the pathogenesis of AD; interestingly, the effect of TSLP on the activation of dendritic cells and the subsequent activation of the Th2 response is well known, but what this research group found is that the effects of TSLP act upon T lymphocytes, switching the immune response from Th1/Th17 to Th2/Th22 without the aid of dendrocytes [42]. Lyubchenko’s team developed a novel way of sampling proteins extractable from the skin of AD, using skin taping. Alongside TSLP, the other isolated interleukins included IL1-b and IL-18, which were both involved in inflammation processes. Furthermore, they were all found to be related to the severity of the SCORAD and loss of water through epidermis [60]. Moon et al. investigated the use of physcion, an anthraquinone, for use as a possible treatment for AD. Samples derived from murine skin, after its administration, demonstrated lower levels of many interleukins involved in the pathogenesis of AD, among which TSLP was arguably the most important. Further studies will be required, but positive results in this sense could validate a new possible treatment for this form of dermatosis [54]. Kim et al. focused on an eastern Asia plant to alleviate AD manifestations and symptoms by blocking the activity of TSLP, leading to contrasting results. One of the most interesting findings is that TSLP acts as a first pruritus mediator in AD lesions via histamine, by eliciting slow neuron fibers able to recognize the itching sensation [50]. Ko et al. explored another plant extract, ginsenoside Rh2 from ginseng, for use as a possible therapeutic method to treat AD; other than dampening the effects of other cytokines such as IL-8 and TNF, its effects on TSLP are exerted by affecting the nuclear factor-kB (NF-kB) pathway [55]. Yoou et al. proposed a traditional Korean salt to ameliorate AD skin lesions and found that the oral administration of bamboo salt in mice lowers levels of TSLP by stimulating IL-32 on monocytes, suggesting that certain foods could improve the condition of these patients [49]. Yeo et al. studied the role of chrysin, a flavonoid present in several foods, as a protective molecule in AD. They found that, in both in vitro and in vivo models of AD, chrysin can downregulate the early growth response (EGR1) expression in HaCaT cells, leading to a downregulation of TNA-alpha and consequent lowering of TSLP production. The lower levels of TSLP led to better clinical conditions in the in vivo branch of this study and to less inflammation in the in vitro branch [61]. Lou et al. studied the various levels of TSLP expression in AD affected children, noticing that one of these variations, rs1898671, is linked to AD developing in African American children, related to food and drug allergy. It is the authors’ opinion that specific variants of TSLP could lead, in the future, to person-tailored target therapies with better outcomes [53]. Wang et al. explored the possibility of targeting a specific micro-RNA, small RNA molecules able to silence other RNA sequences, to decrease expression of TSLP. One of these, miR-155-5p, when blocked, leads to a higher production of filaggrin, lesser production of TSLP and IL-33, which is another interleukin involved in this form of dermatosis, and a strengthening of tight junctions among cells via upregulation of occludin [56]. Guo et al. used TSLP as a marker of disease activity in their study. In this murine model, an AD-like dermatosis was induced to challenge the efficacy of a metal-binding protein with scavenger skills, metallothionein, as a possible protective factor in this condition. Knock out mice for this protein did, indeed, show higher levels of TSLP [48]. Herro et al. present research regarding a cytokine belonging to the TNF superfamily and demonstrated that this protein upregulates the production of TSLP if paired with its receptor. According to the authors’ suggestion, blocking this pathway leads to AD-lesion resolution [51]. Kumagai et al. conducted a study of immunohistochemistry on human skin samples and in vitro cultures of keratinocytes in order to define other means of TSLP production. They found that a p 53-related molecule, ΔNp63, represents a positive feedback stimulus in the production of TSLP, with an autocrine mechanism, previously activated by Toll-like receptor 3 and by the enhanced stimulation of the already cited NF-kB [45]. Mizuno et al. investigated the role of short-chain fatty acids (SCA) in inducing the expression of TSLP in a culture of murine keratinocytes. They found that pentanoic acid is a stronger inducer of TSLP, in a concentration dependent manner. In addition to this they proposed one possible molecular mechanism and, as consequence a possible therapeutic target, of induction of TSLP by pentanoic acid. This involves g protein receptors signaling pathways, including FFAR2 and FFAR3, which are a family of Gq/11- and Gi-coupled receptors [47]. Kim et al. provided an interesting study about the possible influence of the gut microbiome and AD. They found that an oral administration of the prebiotic kestose, a fructooligosaccharides (FOS) in a murine model, suppresses the expression of TSLP, IL-4 and Th-2-related cytokines, thereby ameliorating skin inflammation and providing benefits in clinical response [62]. Gourru-Lesimple et al. studied the link between measles infection (MV) and the development of AD. They found that human keratinocytes express MV receptors and are susceptible to MV infection. They found that MV can modulate the expression of various keratinocyte-produced cytokines, including TSLP, producing a beneficial effect through a lowering of its skin levels. This result suggests the MV vaccine’s possible beneficial role in the prevention of the progression of AD [46]. An original investigation was provided by Chang et al. in which they designed a cohort study of 842 children with atopic dermatitis to define if variations in the filaggrin (FLG) and TSLP genotype are related to differences in the administered treatment. They found that in patients with 2 FLG, the loss of function alleles was less likely able to achieve total skin healing and steroids were more beneficial in treatment, and mutations of TSLP rs1898671 homozygotes were less likely to use calcineurin inhibitors instead of steroids. Among all patients that had discontinued topical calcineurin inhibitors, those with the rs1898671 single-nucleotide polymorphism were more likely to have stopped other treatments, suggesting a somewhat negative role [43].

## 4. Discussion

**Figure 1:**
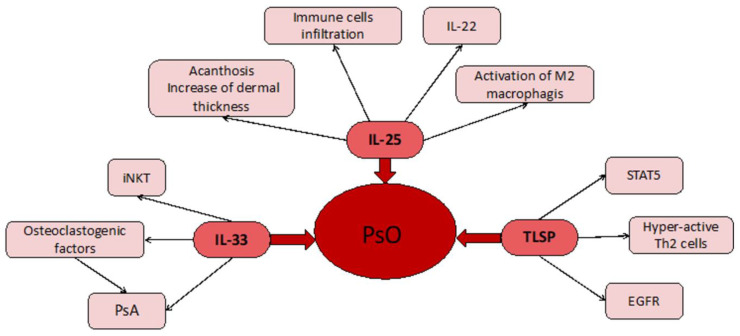
At the center we have the main disease (Pso). This is linked to the three cytokines (TSLP, IL-25, IL-33) used in studies. Depending on the cytokine, we find different manifestations and local responses.

**Figure 2:**
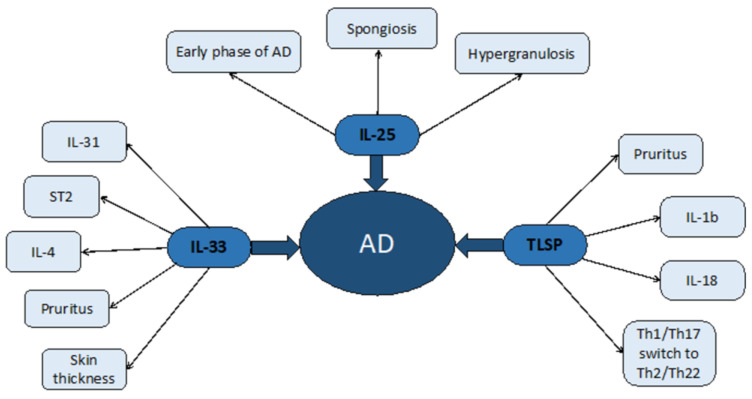
Atopic dermatitis, as in the previous image, has been represented in the middle with the three cytokines examined depicted around it. Secondary effects and symptoms consequent to epithelial cytokines have been represented on the side of the picture.

By analyzing the studies published so far, our research found 51 results. Since the research was based on the possible relationship between epithelial cytokines and common chronic skin conditions, it was plausible to expect approximately the same number of articles relating to Pso and, but this was not the case. In fact, 37 articles were related to these cytokines and AD whereas 14 were concerned with the same cytokines and Pso. This finding is unusual, considering that, at least some cytokines, appear to be more prominently represented in the acute phase of Pso rather than AD [34]., AD has been thoroughly studied in relation to both types of dermatoses, but Pso might represent an interesting field of study. In particular, the role of TNF-alpha should be evaluated, since TSLP appears to be conducive to an increase in TNF-alpha activity [35,37] and several molecules have been used so far to treat Pso by targeting TNF-alpha [63]. It is plausible to suggest that, since TNF-alpha treatments could lead to several side effects, including the development of new Pso lesions [64], targeting a molecule down the same pathway, in this case TSLP, could lead to better results in terms of skin clearance and less side effects. Regarding IL-25 and its role in Pso, it seems that it is involved in the acute phases of this disease, [12,13,14,15], leading to the accumulation of neutrophils, especially the basal layers and is directly responsible of Pso-like lesions in the specimen used. This leads to the observation that targeting this interleukin at the very beginning or acute phases of the disease could lead to rapid ameliorating effects. It is plausible to suggest that these antibodies could be used in targeting IL-25 as a possible therapeutic option for the treatment of severe cases of erythrodermic Pso, where the rapidity of the response is crucial. Unfortunately, only one study was produced relating to this topic, in Europe, where a patient with erythrodermic Pso was treated and reached PASI 100 at the 6th week of treatment with ixekizumab [65], an inhibitor of IL-17A, as previously mentioned, belonging to the same superfamily of IL-17E (IL-25). As for IL-33 and Pso, the four studies taken in consideration led to contrasting results. On one hand, levels of IL-33 were not found to correlate with Pso severity nor did in situ injections produce the appearance of new lesions but, rather, led to a protective role [20,21]; and on the other, Meephansan compared two well established Pso treatments, NB-UVB and MTX, and found that both treatments are efficacious but MTX, as an immunosuppressant, produces low levels of IL-33 whilst light-therapy leads to its increase [19]. Finally, the study involving RANKL, a potent activator of bone reabsorption which is increased via IL-33, suggests a link between psoriatic cutaneous inflammation and the pathogenesis of psoriatic arthritis (PsA), thus explaining why, in most cases, Pso anticipates several decays the onset of PsA [18]. Chen suggested a possible solution to this issue through a new class of T cells, as invariant natural killer T-cells (iNKT) are responsible for the killing of Th17 cells which, paired with Th1 cells, represent the two most common immune system populations involved in the development of new plaques [21]. On the other hand, a possible link between iNKT and osteoclasts was suggested by another study, involving myeloma but not Pso, regarding osteoclasts’ activity [66]. This could explain why the same cytokine, IL-33, may play a protective role in the skin and a detrimental role in the bone, since its presence leads to the expression of RANKL and consequent bone reabsorption [18]. It would be interesting to attempt a topical approach rather than a systemic approach to exploit the effects of IL-33 on the human skin without side effects. On the other hand, in Pso, systemic IL-33 targeting could lead to positive results. In a review by Cannavò et al., several studies led to the conclusion that mast cells are well integrated in Pso pathogenesis and are activated via IL-33 and the pairing with its receptor, which leads to IL-1, IL-6 and IL-13, thus perpetrating the inflammatory effect [67]. Finally, TSLP and Pso, suggest both a pathogenetic and symptomatic role of this cytokine. Both Gago-Lopez and Segawa’s studies demonstrated that this molecule could produce growth factors towards the endothelium and the epidermis, thus explaining two main features of the psoriatic lesions, including thickening and vascular resurfacing [35,38]. Schaper researched the link between histamine release and TSLP in Pso. This could also prove useful since Pso, a condition that has not been associated with itching, may find a possible explanation in this relation regarding this symptom [34]. Moreover, it is arguable that if the Th2 response is indeed causative of some lesions, at least in some patients, there could exist a phenotype of Pso most likely prone to develop itchy lesions and other lesions that are not itchy [27]. Both AD and Pso, then, may provide an intense activation of the inflammatory system and both have recently been found to provide useful insights on the immunological profile and cell subpopulations involved in their development, even when using minimal research methods such as the tape strips method [60,68]. This observation leads us to the conclusion that, even though systemic therapies for this condition are required in more severe cases, novel treatments such as the inhibition of certain miRNAs or the inhibition of oxygen flow could represent an alternative to older, hit-or-miss therapies such as topical steroids, to avoid side effects and could lead to skin clearance [26,30]. In terms of AD, the cytokines studied were IL-33 and TSLP. Research on IL-25 led to just two results, which is not nearly enough to suggest any possible proposition but leads to a need for further studies in this sense, since IL-25 might play a role in the very early phases of this dermatosis. Regarding AD and IL-33, there were no univocal results. Although studies suggest that, in Pso, IL-33 might play a protective role, in AD just three studies out of 12 led to the conclusion that IL-33 might play a secondary role [28,33] or could lead to an expansion of the Treg subpopulation [22], thus stopping the inflammation. On the other hand, nine of the studies either proposed IL-33 or its downward products as a possible target for future therapies [26,31] or suggested a possible role of IL-33 as a causative agent of AD lesions [24,25,27,30,32], especially since pruritus is one of the main components of this condition and IL-33 is intimately linked to IL-31, another cytokine that is currently studied as a potential factor of the itching [69]. In particular, the role of IL-33 in suppressing the expression of crucial barrier function-oriented proteins, such as claudin and filaggrin could be provide the best explanation as to why this cytokine could be involved in the very early phases of this disease [27,30]. Seo proposed an interesting result from his study. The presence of heat-sensitive TRPV3 in AD prone skin could be the primary cause of the production of IL-33 but also TSLP, another pruritogen interleukin [31]. This result might suggest, perhaps, that it is not just the lack of barrier function of the skin due to the loss of filaggrin that is the primary cause of the cascade of events that leads to skin lesions, but rather an endogenous predisposition of the patient to the loss of barrier due to the receptors’ abnormal activation. Interestingly, identifying new targets for future therapies could lead to an earlier resolution and could provide matching for the AD-affected patient. As for TSLP and AD, a high number of recent studies focused on this cytokine as a possible biomarker of disease activity [48] and potential target therapy. Lou et al. suggested that AD does not represent a univocal disease, but rather a complex anthology of variants, each requiring thorough assessment in order to be treated with the best possible outcomes [53]. Severe, hyper-IgE forms might significantly improve with new therapeutic options, such as target therapy using TSLP. Several studies focused on molecules that, when lacking, lead to a spike in its production, such as ZAG, an adipokine which, when missing, leads to lower levels of ceramides and filaggrin production in atopic skin [57]. ZAG, when lacking, leads to an increased production in TSLP, which manifests its effects via skin thickening and itching. TSLP seems to be linked to other pro-inflammatory cytokines such as TNF-alpha, according to Herro [51]. Anti-TNF-alpha treatments are known for their side effects, such as a predisposition to microbial infections, thus targeting the same pathway but at a more specific site rather than generically targeting the inflammatory response and could prevent these patients from having unpleasant side effects. Another interesting field of research is the link between TSLP-production and skin pathogens or resident flora. A study suggests the preventive role of the measles vaccine in order to downregulate TSLP production, but no clear link has been established between AD and virus infections [46]. On the other hand, with regard to bacteria, TSLP has been found to act as a down regulator of antimicrobial peptides, which could explain why the basal levels of this cytokine, that is upregulated in AD affected subjects, leads to severe microbial infections such as eczema herpeticum or the colonization of pre-existing skin lesions [41]. This effect might be enhanced by the same products of microbial agents such as LPS, according to Gu et al., that lead to higher levels of TSLP in the examined skin samples [48]. The external stimulus that triggers skin lesions in AD-prone patients might be represented by the interaction of other pattern recognition-linked molecules, such as Toll-like receptors, closely related to LPS. An autocrine mechanism of TSLP production is triggered through the activation of TLR3, leading to more scratching and increased skin thickening [45] via the NF-kB pathway. On the same note, other microbial products such as pentanoic acid, a fat acid produced by skin bacteria, leads to higher TSLP production, via Gi-protein coupled receptors [47]. All these pathways suggest a crucial role of the skin flora in triggering AD lesions, suggesting that specific subpopulations of microbes present in the most affected sites of this dermatosis could be the cause of the lesions, rather than the classical theory of filaggrin loss in flexural areas. Kim et al. furthered this idea, suggesting that even the gut microbiome might affect cytokine production, especially TSLP and IL-4 and could represent a predisposing factor of this condition [62]. Previous studies led to the conclusion that gut bacteria can affect the skin, such as in cases of acne [70], but studies regarding AD are insufficient. Another studied target is PAR2, a receptor surface which, when stimulated, leads to TSLP production and the blockade of this receptor via a cell-penetrating pepducin, which results in less skin thickening [52]. Moreover, TSLP can be used to assess disease severity according to one study [44], since in AD-prone children, its levels are significantly higher than healthy controls. Furthermore, in a different study, the identification of some TSLP variants were found to lead to treatment resistance for traditional drugs such as corticosteroids [43]. TSLP, according to another study, is crucial in the production of IL-4 in AD models, which leads to a Th2 switch and T CD4+ proliferation in the damaged skin [59]. This switch could be caused by TSLP alone, without the previous activation of dendritic cells [42], which are well known for their importance in AD pathogenesis [71]. Novel therapies might include miRNA blockade [56], possibly administered as a topical product, perhaps in conjunction with narrow band UVB therapy [72]. Finally, the cosmetic and adjunctive treatment options seem to have broadened over the years. Five studies proposed the use of several bioproducts such as salts and plant extracts, in order to ameliorate the red patches in AD by lowering levels of TSLP, underlying the fact that this cytokine is crucial in the itching sensation and skin thickening. All of these new treatments, which are efficacious in the short run, suggest that therapeutic options, at least topically and when paired with broader treatments, could ameliorate these patients’ quality of life [49,50,54,55,61]. All of these studies seem to point in one direction, suggesting that epithelium-derived cytokines play a major role in chronic dermatoses. Oxidative stress may also provide a possible causative agent. Oxidative stress has been identified as an elicitor of TSLP production [73], although this has not been proven in the skin, and IL-33 and IL-25, have been linked to it in other allergic diseases such as asthma [74,75]. The control of oxidative stress in these diseases provided by antioxidants could suggest them to be a helpful tool in treating chronic skin inflammation [76]. AD and Pso represent two diseases in which interleukins play a pivotal role. Some recent studies suggest a common link between these two diseases. Chronic AD lesions, in particular with lichenification and in subsets of Asian patients, have demonstrated that a Th1 response is common and could represent a potential target for biologics in the future, even though currently, no trial is underway [77]. Other factors, such as genes [78] and metabolic pathways [79], have been studied and compared regarding these two diseases but no real conclusion of possible common aspects of the two conditions have been found. Moreover, the same cytokines could play different roles depending on the condition affecting the patient, depending on the presence of AD or Pso. For example, interleukins 31 and 33 play a role in both diseases’ pathogenesis and pruritus but are involved in different pathways [80]. Ongoing clinical trials concerning biological drugs that block these cytokines represent one of the best solutions for these chronic dermatoses, especially when paired with already well-known treatments such as anti-TNF, anti-IL12/23 and anti-IL17 monoclonal antibodies. In this sense, several molecules have been proposed, some of which have been reviewed in this paper. Etokimab is one of such molecules, a monoclonal antibody directed against IL-33. However, no official clinical trials have been proposed against already known gold standards for the above mentioned dermatoses, such as anti-IL-12/23 or anti-IgE, as in the case of psoriasis. Overall, this drug seems to possess a safe profile since the only adverse event identified occurred in a phase 2 clinical trial, which found the worsening of an already ongoing depressive syndrome in a single patient [29]. Another promising drug might be Tezepelumab, directed against TSLP, which showed a greater improvement in treated patients as opposed to those with a placebo, but not dramatically enough to be considered statistically significant, and showing a better control of asthma-affected individuals. It was the author’s opinion that further studies are needed since there are, as of today, no comparative studies meeting the current gold standards for AD treatments [81]. Explicative diagrams of the findings are presented in Figure 1 and Figure 2.

## 5. Conclusions

With the advent of biologic therapies, patients affected by chronic inflammatory skin diseases have experienced an improvement in their quality of life and disease-free time. Two common dermatoses such as atopic dermatitis and psoriasis have benefitted greatly in terms of therapy due to the drugs mentioned in this study, but there is still a great margin for improvement. The epithelium-derived cytokines, namely TSLP, IL-25 and IL-33 represent a new target for future therapies, especially in terms of itch control and, possibly, side-effects minimization, but not enough studies, especially in psoriasis, have been conducted in this direction. In the future, these interleukins will hopefully be validated as representing a therapeutic target for patients worldwide, possibly in conjunction with other available or in-development medications.

## Figures and Tables

**Table 1:** Interleukin 25 and skin. The results include both psoriasis and atopic dermatitis. The results are ordered by year and a quick overview is presented by author’s name, disease studied, species examined and results.

Author	Disease Studied	Species Examined	Year	Results
Xu et al. [12]	Pso	Animals	2018	IL-25 levels are increased in psoriatic skin. IL-25 knockout mice show lower amounts of acanthosis, thickness and immune cell infiltration
Senra et al. [13]	Pso	Animals	2019	IL-25 leads to an accumulation of neutrophils, led by macrophages, at the site of injection
Borowczyk et al. [14]	Pso	Colture cells	2020	IL-22 upregulates the production of IL-25
Senra et al. [15]	Pso	Colture cells	2016	In basal and suprabasal levels of epidermis of Pso lesions, levels of IL-25+ cells are higher than in controls
Yi et al. [16]	AD	Colture cells	2017	Intelectin high levels in skin samples of AD without upregulation of IL-25
De Vuyst et al. [17]	AD	Colture cells	2018	Cells incubation with IL-25 causes spongiosis and hypergranulosis

**Table 2:** Interleukin 33 and skin. Results are inclusive of both psoriasis and atopic dermatitis. The results are ordered by year and a quick recap was produced by author’s name, disease, specimens and results.

Author	Disease Studied	Species Examined	Year	Results
Raimondo et al. [18]	Pso	Humans	2017	IL-33 from psoriatic plaques induces the release of pro-osteoclatogenic factors
Meephansan et al. [19]	Pso	Humans	2018	Decrease of IL-33 levels after treatment with MTX, increase after NB-UVBtreatment
Borsky et al. [20]	Pso	Humans	2020	High sera levels in psoriatic patients, no correlation with the severity of clinical presentation
Chen et al. [21]	Pso	Humans	2020	High sera and intraepidermal levels of IL-33 in patients with moderate-severe psoriasis
Salamon et al. [22]	AD	Colture cells, animals	2017	IL-33 induces a production of IL-2
Jang et al. [23]	AD	Colture cells	2017	HDM induces the release of IL-33 and IL-25
Nygaard et al. [24]	AD	Colture cells	2017	Down regulation of members of the epidermal differentiation complex after exposure to IL-33
Tang et al. [25]	AD	Animals	2018	Knock down of SHAPIN causes AD via IL-33
Peng et al. [26]	AD	Animals	2018	Antibodies anti-IL33 lead to reduction of lichenification, redness and scaling
Ryu et al. [27]	AD	Colture cells	2018	Low levels of claudin-1 after exposure to high levels of IL-33
Sawada et al. [28]	AD	Animals	2019	Increased levels of IL-33 in the model of disease
Chen et al. [29]	AD	Humans	2019	Etokimab: Good tolerability and efficacy (EASI score)
Nakamura et al. [30]	AD	Humans	2019	Correlation with levels of IL-33 and degree of lichenification and pruritus
Seo et al. [31]	AD	Humans	2020	High TRPV3 levels in AD TRPV3 induces the production of IL-33
Dai et al. [32]	AD	Colture cells	2020	Dermatophagoides pteronissynus allergens induce overexpression of IL-33
Pietka et al. [33]	AD	Animals	2020	Wild type and knockout mice for IL-33 and its receptor develop AD lesions after treatment with calcipotriol

**Table 3:** TSLP and skin. Results include psoriasis and atopic dermatitis. The results are ordered by year and a quick recap was produced by author’s name, disease, specimens used and results.

Author	Disease Studied	Species Examined	Year	Results
Schaper et al. [34]	Pso	Colture cells	2016	Pso patients present high levels of inflammation-related isoform of TSLP
Segawa et al. [35]	Pso	Colture cells	2017	EGFR is transactivated via TSLP by TNF-alpha
Desmet et al. [36]	Pso	Colture cells	2018	miRNA against TSLP topically used decreases the release of TSLP
Tashiro et al. [37]	Pso	Colture cells	2019	TSLP presents reduced levels in hypoxic conditions
Gago-Lopez et al. [38]	Pso	Animals	2019	Antibodies anti-TSLP lead to a positive response in epidermal thickening and vascularization
Chen et al. [39]	Pso	Humans	2021	Early onset patients have high levels of Th2 cells and TSLP
Bogaczewicz et al. [40]	AD	Humans	2015	UV-A therapy does not lower TSLP serum levels
Lee et al. [41]	AD	Colture cells	2016	TSLP acts as a downregulator of the AMPs via the JAK/STAT3 pathway
Wallmeyer et al. [42]	AD	Animals	2017	TSLP acts upon T lymphocytes switching the immune response from Th1/Th17 to Th2/Th22 without the aid of dendrocytes
Chang et al. [43]	AD	Humans	2017	Patients with mutation of TSLP rs1898671 homozygotes are less likely to use calcineurin inhibitors instead of steroids
Uysal et al. [44]	AD	Humans	2017	Higher levels of TSLP in the blood correlate to severity of clinical manifestations
Kumagai et al. [45]	AD	Colture cells	2017	ΔNp63 is a positive feedback stimulus in the production of TSLP
Gourru-Lesimple et al. [46]	AD	Colture cells	2017	MV modulate the expression of TSLP
Mizuno et al. [47]	AD	Colture cells	2017	Pentanoic acid is a potent inducer of TSLP
Guo et al. [48]	AD	Animals	2018	Metallothionein knock down mice show higher levels of TSLP
Yoou et al. [49]	AD	Animals	2018	Bamboo salt lowers levels of TSLP by stimulating IL-32 on monocites
Kim et al. [50]	AD	Animals	2018	Kestose oral administration suppresses TSLP expression
Herro et al. [51]	AD	Animals	2018	Tumor necrosis factor (TNF) superfamily protein LIGHT (homologous to lymphotoxin), exhibits inducible expression and competes with HSV glycoprotein D for binding to HVEM, (a receptor expressed on T lymphocytes) upregulates the TSLP production
Barr et al. [52]	AD	Animals	2018	PZ-235 treatment reduces thickening of AD lesions
Lou et al. [53]	AD	Humans	2019	rs1898671 variant of TSLP is linked to AD developing in African American children and related to food and drug allergy
Moon et al. [54]	AD	Animals	2019	Anthraquinone treatment reduces TSLP levels
Ko et al. [55]	AD	Animals	2019	Ginsenoside Rh2 damps the effects of TSLP by affecting NF-kB
Wang et al. [56]	AD	Animals	2019	Block of miR-155-5p leads to higher production of filaggrin, lesser production of TSLP and IL-33
Noh et al. [57]	AD	Humans	2019	ZAG regulates TSLP secretion
Gu et al. [58]	AD	Colture cells	2020	Bland topical treatments turn down the expression of TSLP
Kitajima et al. [59]	AD	Animals	2020	Blockade of the receptor of TSLP (TSLPR) in CD4+ lymphocytes interrupts the chronic phase of inflammation
Lyubchenko et al. [60]	AD	Humans	2020	TSLP isolated from AD patients’ skin
